# 

**Published:** 1888-12

**Authors:** 


The Chicago Medical Journal and Examiner.

Volume 57. Number 6.

### CONTENTS OF THIS NUMBER.

**Original Communications—** PAGE.

The Pathology of Joint and Bone Tuberculosis. W. Van Hook ........ 322
The Influence of the Work of The Illinois Medical-Practice Act upon Medical Education. II. A. Johnson ........ 327
Report of a Case of Gastrostomy. B. Holmes ........ 330
Grafting of Skin taken from Fowls, with Report of a Case. B. F. Rogers ........ 333

**Editorial—**

Ptomaïns and their Effects ........ 334
Useful Memorials ........ 335

**Society Reports—**

Chicago Pathological Society ........ 335
Transactions of the Gynæcological Society of Chicago ........ 340
American Academy of Medicine ........ 349
Allegheny County Medical Society ........ 352
American Public Health Association ........ 356

**Foreign Correspondence—**

Italian Medical Congress. A. Lagorio ........ 359

**Obituary—**

Dr. H. D. Schmidt ........ 361

Professor H. B. Sands, M. D. ........ 362

**Extracts and Abstracts** ........ 363
**Book Reviews and Notices** ........ 376
**Books Received** ........ 377
**Pamphlets Received** ........ 378
**Index to Advertisers** ........ x

